# Relationship between advanced lung cancer inflammation index and all-cause and cause-specific mortality among chronic inflammatory airway diseases patients: a population-based study

**DOI:** 10.3389/fimmu.2025.1585927

**Published:** 2025-05-15

**Authors:** Zhuanbo Luo, Peixu Chen, Shiyu Chen, Xue Kong, Hongying Ma, Chao Cao

**Affiliations:** Department of Respiratory and Critical Care Medicine, Key Laboratory of Respiratory Disease of Ningbo, The First Affiliated Hospital of Ningbo University, Ningbo, Zhejiang, China

**Keywords:** advanced lung cancer inflammation index, chronic inflammatory airway diseases, mortality, population-based study, NHANES

## Abstract

**Background:**

Chronic inflammatory airway diseases (CIAD), such as asthma, chronic bronchitis, and chronic obstructive pulmonary disease, pose a significant threat to public health, with its prognosis closely tied to the body’s inflammation level and nutritional status. As a composite indicator, the advanced lung cancer inflammation index (ALI) integrates inflammation and nutritional status. Despite its potential utility, the link between ALI and the prognosis of patients with CIAD remains unexplored. This study aimed to investigate this relationship.

**Methods:**

We gathered data from the National Health and Nutrition Examination Survey (NHANES) from 2013 to 2018. The National Death Index was used to calculate mortality until December 31, 2019. Kaplan-Meier analysis was employed to investigate the relationships between ALI and all-cause and cause-specific mortality in patients with CIAD. Furthermore, weighted univariable and multivariable Cox proportional hazards models were employed to further examine their relationship. Multiple factors that could impact the results were adjusted in the analysis. We also utilized a restricted cubic spline analysis to estimate the non-linear relationships between ALI and all-cause and cause-specific mortality rates in patients with CIAD. Finally, subgroup and sensitivity analyses were conducted to ensure the reliability of the findings.

**Results:**

The study involved 2,884 CIAD patients. An elevated ALI was significantly related to a decreased risk of all-cause mortality, as well as mortality from cardiovascular and respiratory diseases among CIAD patients. Results from RCS analysis revealed a reverse J-shaped non-linear association between ALI and all-cause mortality in CIAD patients, with an inflection point at 99 (p for nonlinearity <0.0001). The inflection point in the J-shaped relationship represents the ALI value with the lowest risk of mortality. For ALI values below 99, a 10-unit rise in ALI was linked to a 14% reduction in the risk of all-cause mortality (HR: 0.86, 95% CI:0.81-0.92, Ptrend=0.01). Conversely, if ALI exceeded 99, a 10-unit increase in ALI resulted in a 3% rise in the risk of all-cause mortality (HR: 1.03, 95% CI:1.01-1.06, Ptrend=0.02). A similar J-shaped association was observed in mortality due to cardiovascular and respiratory diseases, with inflection points at 94 and 96, respectively. These findings were consistent across sociodemographic and prior disease-related subgroups, and remained stable in sensitivity analyses.

**Conclusion:**

This study revealed a novel association between elevated ALI level and reduced all-cause and cause-specific mortality risk in patients with CIAD. Furthermore, the relationship between ALI and mortality rates from all cause, as well as cardiovascular and respiratory diseases, exhibited an non-linear, J-shaped curve. These findings underscore the importance of maintaining optimal ALI levels within a specific range to promote long-term survival in CIAD patients. The dynamic variation in ALI over time also can help clinicians establish personalized ALI standards to maximize the long-term outcomes for CIAD patients.

## Introduction

For decades, chronic inflammatory airway diseases(CIAD), such as asthma, chronic bronchitis, and chronic obstructive pulmonary disease (COPD), have been a major public health concern (1). The increasing prevalence of respiratory diseases has been linked to environmental factors, lifestyle choices, and demographic shifts, including industrialization, urbanization, and an aging population, resulting in a steady rise in both incidence and mortality rates. In 2017, chronic respiratory diseases were the third leading cause of death globally, accounting for 7.0% of all fatalities, with asthma and COPD being the most prevalent chronic respiratory conditions (1). According to recent data, asthma affected over 260 million people worldwide, resulting in 455,000 deaths due to inadequate management (2), while COPD claimed 3.3 million lives among 212.3 million reported cases (3).

The pathogenesis of CIAD is complex and influenced by various environmental and genetic factors (4, 5). Systemic inflammation and immune response play a significant role in the progression of chronic airway inflammation, acting as a crucial pathogenic mechanism (6, 7). Several types of inflammatory cells are drawn to and become active in the airways, contributing to the body’s inflammatory and immune reactions (4). Prior researches have demonstrated that the presence of inflammatory cells can cause harm to structural cells in the airways, such as airway epithelial cells, matrix cells, and parenchymal cells and the severity of COPD is linked to the level of airway inflammation (8). In asthma, airway inflammation is intensified by specific immune responses involving Th2 cells, IgE-producing B cells, mast cells, and eosinophils (9). Alongside the adaptive immunity triggered by allergens and Th2 cells, innate immune cells like macrophages, granulocytes, epithelial cells, mast cells, eosinophils, dendritic cells, and natural killer cells are believed to contribute to the development of asthma, both in allergic and nonallergic contexts (9). Neutrophils play a crucial role in the pathogenesis of CIAD, serving as the frontline defense during lung infections by releasing chemokines and granulins that attract monocytes and/or macrophages to the infection site, leading to an immune response (10, 11). An elevated count of neutrophils in the blood is a hallmark of all CIAD patients. Previous researches have indicated that higher levels of inflammatory markers, such as the neutrophil-to-lymphocyte ratio (NLR), may be linked to a greater risk of worsening symptoms and mortality in patients with COPD (12, 13). A meta-analysis by Huang et al. found that the NLR is a useful and easily measurable indicator for assessing asthma and its severity (14). Additionally, prolonged chronic inflammation can lead to insulin resistance, triggered by inflammatory factors such as TNF and C-reactive protein (CRP), resulting in weight loss and decreased albumin levels (15, 16). Previous studies have shown that nutritional indicators, including albumin and body mass index (BMI), are closely tied to the prognosis of CIAD patients (17, 18). As a result, inflammation can impact the prognosis of CIAD patients both directly and indirectly through its effects on albumin and BMI. Therefore, relying solely on inflammation markers to predict the prognosis of CIAD patients may be insufficient, highlighting the need for new, comprehensive indicators to evaluate patient outcomes.

Jafri and colleagues introduced the advanced lung cancer inflammation index (ALI) in 2013 as a novel prognostic tool for individuals with advanced lung cancer (19). This index is a combination of BMI, albumin levels, and NLR, providing insights into the inflammatory and nutritional status of the patients. Initially developed to forecast tumor outcomes in lung cancer patients, numerous studies have demonstrated its significant predictive capability for the prognosis of individuals with lung cancer (20, 21). Additionally, ALI has shown promise in predicting outcomes across various conditions such as gastric cancer (22), diabetes (23), heart failure (24), and B-cell lymphoma (25). However, limited research has explored the correlation between ALI and the prognosis of patients with CIAD.

Hence, in order to fill this void, our research drew on data from 2,884 individuals aged 20 and above from the national health and nutrition examination survey (NHANES) database (2013-2018) to explore the relationship between ALI and the risk of mortality from all causes and specific causes in patients with CIAD, with the ultimate goal of informing treatment and management strategies for CIAD patients.

## Materials and methods

### Study population

NHANES is a survey conducted in the United States by the National Center for Health Statistics, aiming to represent the nation. It utilizes a stratified multistage random sampling methodology. The research was ethically reviewed by the National Center for Health Statistics (NCHS), and participants all consented to be part of the study. All procedures in this study adhere to relevant rules and standards. NHANES data are accessible on the NHANES website at https://www.cdc.gov/nchs/nhanes.htm.

**Figure 1:**
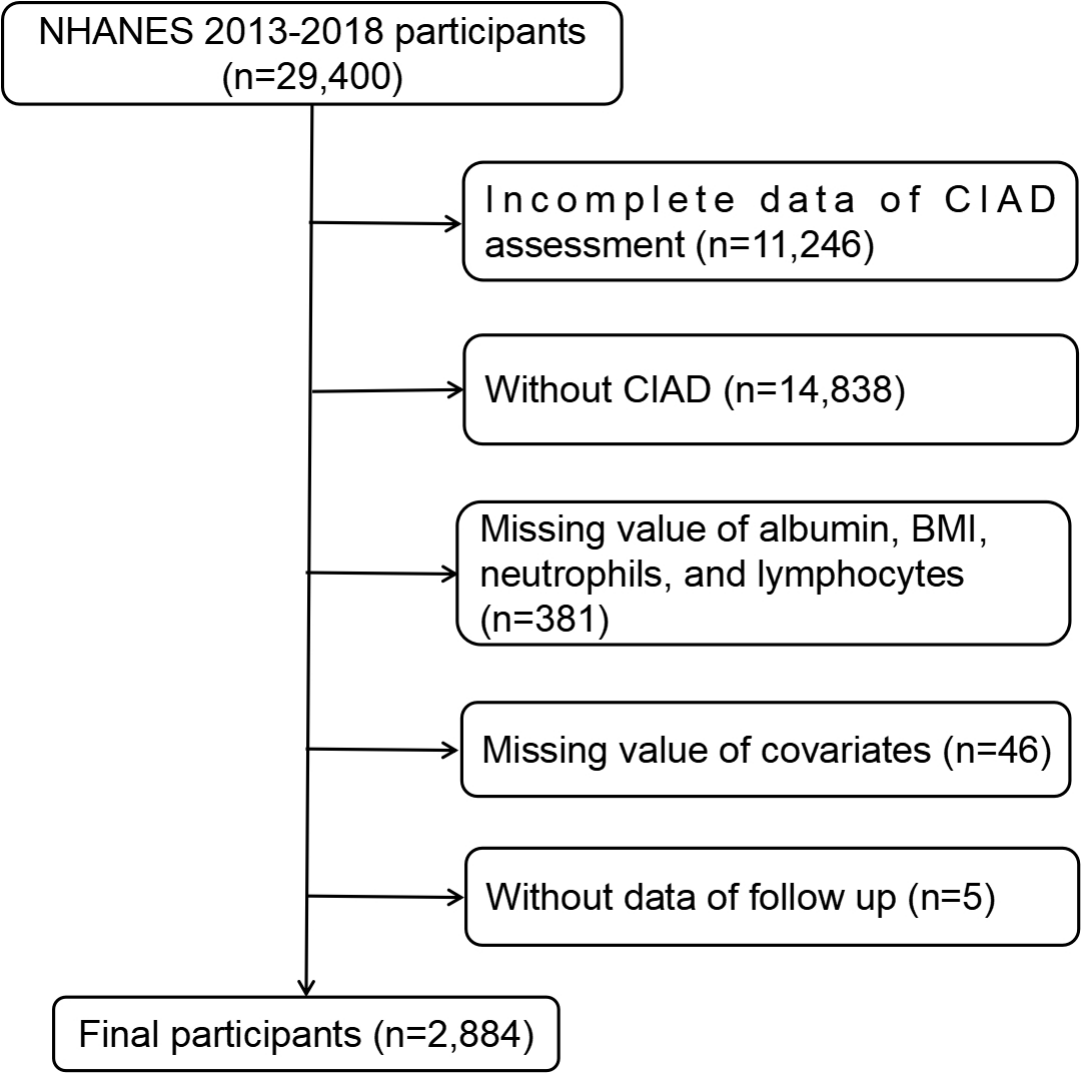
Flowchart of the study participants. BMI, body mass index; CIAD, chronic inflammatory airway diseases; NHANES, national health and nutrition examination survey.

In this research, we analyzed NHANES data spanning from 2013 to 2018, involving a total of 29,400 individuals. Our focus was on adults aged 20 years and above who had been diagnosed with CIAD and had complete information on ALI, along with available mortality data. Initially, we gathered data from 29,400 participants. Subsequently, 11,246 participants lacking CIAD assessment data were removed, and 14,838 participants were excluded without CIAD. Additionally, 381 participants were excluded due to missing information on albumin, BMI, neutrophils, and lymphocytes. Furthermore, 5 participants were omitted for not having data on follow-up time and survival status, and 46 individuals lacked information on covariates. Ultimately, a total of 2,884 eligible individuals were chosen for the study. The detailed procedure of participant selection, along with the inclusion and exclusion criteria, can be found in **Figure 1**.

### Determination of ALI

The ALI score was calculated using a combination of three factors: albumin levels (Alb), body mass index (BMI), and the neutrophil-to-lymphocyte ratio (NLR). Specifically, the score was determined by multiplying albumin levels (measured in grams per deciliter) and BMI (calculated as weight in kilograms divided by the square of height in meters) and then dividing by the NLR (calculated as the ratio of neutrophil counts to lymphocyte counts) (ALI=Alb*BMI/NLR) (26). Patients with CIAD were divided into four groups based on their ALI levels: Quantile 1, Quantile 2, Quantile 3 and Quantile 4.

### Assessment of CIAD

This study defined CIAD as a self-reported asthma, COPD, and chronic bronchitis. Participants were inquired about previous diagnoses of asthma, COPD, or chronic bronchitis made by a healthcare provider. Those who confirmed having received a diagnosis of any of these conditions were categorized as having the corresponding disease. A CIAD diagnosis was assigned to individuals who reported having at least one of these respiratory conditions.

### Assessment of mortality

The research identified the mortality rate as the primary outcome, encompassing death from all causes and two specific causes of death. Cause-specific mortality was classified according to the Tenth Revision of the International Classification of Diseases (ICD-10). This included mortality attributed to cardiovascular diseases (coded I00-I09, I11, I13, I20-I51, and I60-I69) and respiratory diseases (coded J09-J18, J40-J47), using data sourced from the NHANES linked Public-Use Linked Mortality Files until December 31, 2019. Individuals coded as MORTSTAT=0 were assumed to have survived until the end of 2019. The study’s observation period commenced on the date of NHANES enrollment and ended either on the date of death or on December 31, 2019, for those who remained alive.

### Definitions of covariates

To minimize the impact of confounding bias, we carefully selected relevant factors based on clinical relevance and previous studies. We gathered baseline data on participants through questionnaires and physical exams, including demographic information such as age (20-40, 40-60, or over 60), sex (male or female), educational level (more than high school, high school graduate, or less than high school), racial and ethnic identity (Mexican American, non-Hispanic white, non-Hispanic black, or other), and body mass index (BMI) (<25.0, 25.0-29.9, or >30.0 kg/m2). In addition, we evaluated socioeconomic status by calculating the poverty-income ratio (PIR), which compares a family’s income to the poverty threshold for their household size as specified by the US Department of Health and Human Services. We then grouped these ratios into three distinct categories: below 1.3, between 1.3 and 3.5, and above 3.5 (27). Marital status was grouped into three categories: married or living with a partner, widowed or divorced, or never married. Participants’ smoking habits were divided into three categories: never smoker (smoked fewer than 100 cigarettes in their lifetime), former smoker (smoked over 100 cigarettes but no longer smoke at all), and current smoker (had smoked over 100 cigarettes in life and currently smoke)) based on the self-reported number of cigarettes smoked in their lifetime (28). Their drinking status was categorized as never drinker (had less than 12 drinks in their lifetime), former drinker (had at least 12 drinks in one year but did not drink in the last year, or had not drank in the last year but had at least 12 drinks in their lifetime), current heavier drinker (consuming three or more drinks per day for females, four or more drinks per day for males, or engaging in binge drinking (four or more drinks on same occasion for females, five or more drinks on same occasion for males) on 5 or more days per month), or current mild/moderate drinker (consuming two or fewer drinks per day for females, three or fewer drinks per day for males, or engaging in binge drinking on two or fewer days per month) (29, 30).

Medical status variables that were considered in the study included hypertension, cardiovascular disease (CVD) and diabetes mellitus (DM). Hypertension was determined by a systolic blood pressure of 130 mmHg or higher, a diastolic blood pressure of 80 mmHg or higher, or the use of medication to manage blood pressure (31). Diabetes was identified through a doctor’s diagnosis, or a glycated hemoglobin level of over 6.5%, or a random blood glucose level of 11.1 mmol/L or higher, or a two-hour oral glucose tolerance test result of 11.1 mmol/L or higher, or the use of diabetes medication or insulin (22). Individuals who had been diagnosed by a doctor with conditions like coronary heart disease, heart attack, heart failure, or angina were classified as having cardiovascular diseases (31). Additionally, data on blood cell counts and serum albumin levels, which were needed to calculate the ALI, were retrieved from the database.

### Statistical analysis

The study’s statistical analysis was performed using R software (version 4.3.3), with statistical significance defined as a p-value below 0.05. The analysis adhered strictly to NHANES recommendations, considering sample weights, clustering, and stratified analysis. Normally distributed continuous variables were presented as means and standard error (SE), while non-normally distributed continuous variables were displayed as medians and interquartile ranges. Categorical variables were represented as numbers (percentages). The chi-square test and one-way ANOVA were utilized to detect significant differences in categorical and continuous variables between groups, respectively.

Kaplan-Meier analysis was employed to investigate the relationships between ALI and all-cause and cause-specific mortality in patients with CIAD. Log-rank tests were used to compare survival rates among four groups of participants categorized by quartiles of ALI levels. Weighted univariable and multivariable Cox proportional hazards models were utilized to further examine the association of ALI with all-cause and cause-specific mortality in CIAD adults. Multiple factors that could impact the results were adjusted in the analysis. The hazard ratio (HR) and its 95% confidence interval (CIs) were calculated. Three models were developed: Crude Model without adjustments; Model 1 adjusted for gender, age, and ethnicity; and Model 2, which was adjusted for education level, family income-to-poverty ratio, smoking and drinking habits, marital status, BMI, and medical history of hypertension, cardiovascular disease, and diabetes in addition to the factors in Model 1 was built to provide a comprehensive analysis.

We utilized a combination of methods including restricted cubic spline analysis and multivariate-adjusted Cox regression to examine the non-linear connections between ALI and all-cause and cause-specific mortality rates in patients with CIAD. In instances where the relationship was not linear, we employed a recursive algorithm to pinpoint the inflection points. Additionally, we applied piecewise Cox regression models to delve deeper into the impact of specific thresholds on the data. To assess the effect of a 10-unit change in ALI on all-cause and cause-specific mortality in CIAD patients, we divided each participant’s ALI by 10 and included it as a continuous factor in a multivariable Cox regression analysis. This analysis examined the effect of a 10-unit change in ALI on mortality rates due to all causes and cardiovascular and respiratory diseases in individuals with CIAD. This approach aims to better quantify the impact of changes in ALI on the prognosis of patients with CIAD, providing a more detailed understanding of how changes in ALI affect the prognosis of CIAD patients (both quartile ALI and per 10-unit ALI increment). Additionally, the findings can help healthcare providers dynamically assess the prognosis of CIAD patients and make informed decisions based on their ALI levels.

To ensure the reliability of our study, we conducted sensitivity analyses by performing stratified and interaction analyses to explore potential interactions between ALI levels and stratified variables. Additionally, participants who passed away within the initial two years of follow-up were excluded to minimize the risk of reverse causality bias. Furthermore, the study was restricted to individuals aged 45 and older to investigate the relationship effectively. Finally, after removing patients with a history of cancer at the beginning of the study, we reassessed the associations between ALI and mortality risks in CIAD patients.

## Results

### Baseline characteristics

Data from three consecutive two-year cycles of NHANES (2013-2018) was analyzed, with baseline characteristics of participants grouped by ALI quartiles presented in **Table 1**. The study population had a mean age of 56.24 ± 13.6 years, with a slight majority being female (54.16%), and predominantly identified as non-Hispanic white (69.24%). The mean ALI score was 69.52.

**Table 1:** Cohort characteristics at baseline for study participants according to quartiles of ALI.

Variable	Total (N=2884)	ALI				Pvalue
		Q1(N=721)	Q2(N=721)	Q3(N=721)	Q4(N=721)	
		[2.89,45.92]	(45.92,63.64]	(63.64,87.68]	(87.68,852.98]	
**Age, %**						< 0.001
20-40	886(34.54)	166(27.19)	222(32.93)	245(38.73)	253(39.79)	
40-60	963(35.00)	205(33.55)	244(36.76)	238(33.06)	276(36.88)	
>60	1035(30.47)	350(39.26)	255(30.31)	238(28.21)	192(23.33)	
**Sex, %**						0.02
Female	1630(54.16)	366(57.45)	402(58.61)	415(53.42)	447(50.55)	
Male	1254(45.84)	355(42.55)	319(41.39)	306(46.58)	274(49.45)	
**Family PIR, %**						0.22
<1.3	692(24.54)	166(25.17)	154(23.24)	173(24.71)	199(25.13)	
1.3-3.5	980(34.02)	245(34.85)	243(31.95)	238(33.34)	254(36.06)	
>3.5	1212(41.45)	310(39.97)	324(44.81)	310(41.95)	268(38.81)	
**Body mass index, %**						< 0.001
<25.0 kg/m2	905(34.34)	259(41.16)	256(33.76)	239(29.76)	191(23.80)	
25.0-29.9 kg/m2	977(33.20)	209(32.65)	258(34.56)	245(31.46)	245(38.43)	
>30.0 kg/m2	1002(32.46)	153(26.19)	207(31.68)	237(38.78)	285(37.77)	
**Race/ethnicity, %**						< 0.001
Mexican American	274(5.52)	64(5.20)	53(3.94)	88(6.63)	69(6.45)	
non-Hispanic black	637(11.28)	81(4.96)	136(8.92)	155(10.48)	265(22.34)	
non-Hispanic white	1334(69.24)	421(76.57)	350(72.78)	327(70.03)	236(55.59)	
others	639(13.96)	155(13.28)	182(14.35)	151(12.86)	151(15.62)	
**Marital status, %**						0.25
married/living with partner	1545(58.53)	371(56.76)	414(61.63)	394(59.71)	366(55.38)	
never married	559(18.97)	121(17.33)	140(18.60)	138(18.63)	160(21.73)	
widowed/divorced	780(22.50)	229(25.92)	167(19.77)	189(21.66)	195(22.89)	
**Education level, %**						0.08
Completed high school	691(24.99)	183(26.74)	162(25.11)	172(23.44)	174(24.70)	
Less than high school	576(12.84)	165(14.93)	159(13.98)	123(9.33)	129(13.33)	
More than high school	1617(62.18)	373(58.32)	400(60.91)	426(67.22)	418(61.97)	
**Smoking status, %**						0.03
former	813(28.99)	240(31.93)	183(26.02)	196(30.00)	194(28.00)	
never	1322(45.16)	276(37.88)	336(48.04)	349(46.09)	361(48.87)	
now	749(25.85)	205(30.18)	202(25.94)	176(23.91)	166(23.12)	
**Drinking status, %**						< 0.001
Current heavier drinker	325(10.43)	126(14.29)	90(10.68)	82(9.99)	27(9.12)	
Current light/moderate drinker	1672(58.55)	355(56.24)	418(58.22)	447(57.68)	452(57.85)	
Former	282(9.83)	75(9.62)	69(9.63)	63(11.85)	75(11.52)	
Never	605(21.19)	165(18.85)	144(21.47)	129(20.48)	167(21.51)	
**CVD**						0.01
No	2303(83.01)	607(85.81)	596(84.59)	577(83.99)	523(78.71)	
Yes	581(16.99)	114(14.19)	125(15.41)	144(16.01)	198(21.29)	
**DM**						0.04
No	2174(80.15)	550(82.13)	547(81.96)	551(80.94)	526(78.48)	
Yes	710(19.85)	171(17.87)	174(18.04)	170(19.06)	195(21.52)	
**Hypertension**						0.06
No	1395(53.71)	325(51.92)	363(54.76)	373(56.33)	334(51.31)	
Yes	1489(46.29)	396(48.08)	358(45.24)	348(43.67)	387(48.69)	
Albumin, g/dL	4.19(3.01,5.56)	4.13(4.01,5.23)	4.19(3.42,5.74)	4.23(3.62,5.76)	4.39(3.32,6.01)	0.02
White blood cell(1000 cell/mL)	6.90 (0.09)	8.65(0.13)	7.67(0.10)	5.98(0.08)	4.55(0.12)	0.01
Neutrophil count (1000 cell/mL)	4.79(0.05)	5.80(0.10)	4.76(0.08)	4.18(0.07)	3.24(0.07)	< 0.001
Lymphocyte count (1000 cell/mL)	2.16(0.03)	1.69(0.03)	2.11(0.03)	2.34(0.03)	2.96(0.07)	< 0.001
Platelet count (1000 cell/mL)	259.92(1.23)	215.58(1.64)	245.01(2.06)	268.99(2.06)	303.67(2.50)	< 0.001
NLR	2.53(2.41,2.54)	4.11(3.95,4.25)	2.48(2.35,2.52)	1.97(1.92,2.09)	1.40(1.32,1.47)	< 0.001
ALI	69.52(45.66,82.39)	33.10(21.40.48.86)	54.71(36.30,70.47)	74.17(54.32,95.41)	123.54(103.66,144.58)	< 0.001

ALI, advanced lung cancer inflammation index; NLR, neutrophil to Lymphocyte ratio; PIR, family poverty-to-income ratio; DM, diabetes mellitus; CVD, cardiovascular disease; Q1, Quartile 1; Q2, Quartile 2; Q3, Quartile 3; Q4, Quartile 4.

Notable differences emerged when comparing the Q2, Q3, and Q4 groups to the Q1 group: these groups tended to be younger, with higher BMI value, a greater proportion of males, and a larger percentage of non-Hispanic black individuals. They also had a higher percentage of never-smokers, lower rates of current heavier alcohol use, and higher rates of former and never alcohol use. Laboratory tests revealed that they tended to have higher levels of albumin, lymphocytes, platelets, and ALI, and lower levels of white blood cells, neutrophils, and NLR. Additionally, the prevalence of diabetes and cardiovascular disease elevated as ALI levels increased. Other characteristics, such as PIR, marital status, education level, and hypertension status, remained relatively consistent across groups, as shown in **Table 1**.

### Kaplan-Meier analysis

**Figure 2:**
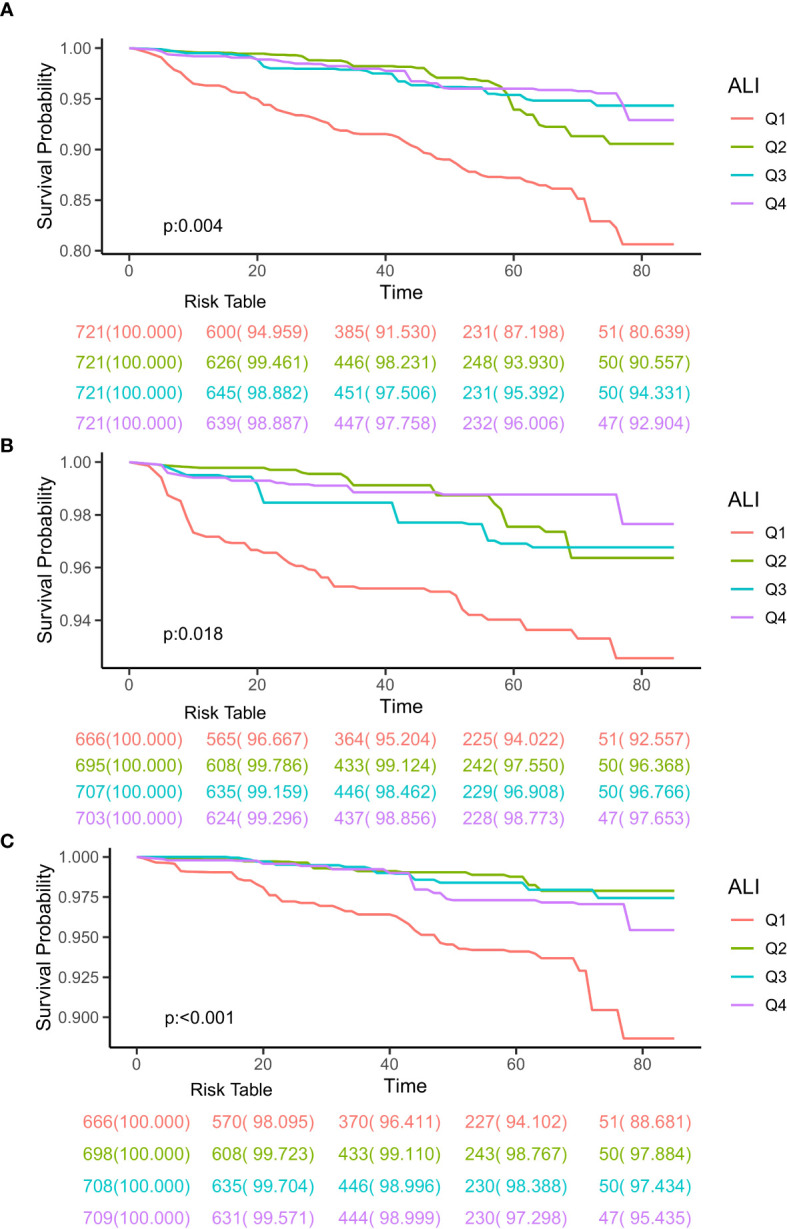
Kaplan-Meier survival curves of ALI impact on long-term all-cause **(A)**, respiratory diseases **(B)** and cardiovascular diseases **(C)** mortality in patients with chronic inflammatory airway diseases (CIAD) categorized by quartiles of advanced lung cancer inflammation index (ALI) levels. Q1, Quantile 1; Q2, Quantile 2; Q3, Quantile 3.

The Kaplan-Meier analysis was used to initially differentiate the connection between ALI and the mortality rates for all causes and specific causes among CIAD patients. During a median follow-up of 3.27 years, there were a total of 196 deaths from all causes, with 93 related to cardiovascular diseases and 83 linked to respiratory diseases. The findings from the Kaplan-Meier survival analysis indicated that individuals in the top quartile of ALI had the least risk of mortality from all causes (log-rank test p = 0.004; **Figure 2A**), respiratory diseases (log-rank test p = 0.018; **Figure 2B**), and cardiovascular diseases (log-rank test p < 0.001; **Figure 2C**) among CIAD patients.

### ALI and mortality

**Table 2** showed the results of Cox regression analysis that investigating the association between ALI and mortality rates from all-cause and cardiovascular and respiratory diseases in CIAD adults. Both crude and multivariate adjusted models revealed a significant inverse relationship between higher ALI levels and reduced all cause mortality in individuals with CIAD. In comparison to the Q1 group, the HR (95% CI) for the Q2, Q3, and Q4 groups were 0.36(0.22-0.60), 0.40(0.22-0.76), and 0.42(0.20-0.90), respectively (P trend=0.03) in the fully adjusted model (Model 2). Similarly, elevated ALI levels were found to be a key predictor of reduced mortality from cardiovascular diseases, with HR (95% CI) of 0.25(0.14-0.43), 0.35(0.17-0.68), 0.56(0.18-0.71), for the Q2, Q3, and Q4 groups, respectively (P trend<0.001) in Model 2. A similar pattern was observed for mortality from respiratory diseases, with higher ALI levels associated with a significantly lower risk of death in CIAD participants. The HR (95% CI) for the Q2, Q3, and Q4 groups were 0.29(0.12-0.67), 0.52(0.23-0.77), 0.31(0.10-0.93), respectively (P trend=0.02).

**Table 2:** Relationships of ALI with all-cause and cause-specific mortality in patients with CIAD from the NHANES 2013–2018 cohort.

	All cause mortality					
	Crude model		Model 1		Model 2	
ALI	HR, 95%CI	P	HR, 95%CI	P	HR, 95%CI	P
Q1	ref		ref		ref	
Q2	0.34(0.19,0.60)	<0.001	0.40(0.23,0.67)	<0.001	0.36(0.22,0.60)	<0.001
Q3	0.30(0.17,0.54)	<0.001	0.37(0.20,0.71)	0.003	0.40(0.22,0.76)	0.005
Q4	0.28(0.14,0.56)	<0.001	0.41(0.20,0.87)	0.02	0.42(0.20,0.90)	0.03
Per 10U increment	0.86(0.74,0.88)	<0.001	0.75(0.72,0.87)	<0.001	0.95(0.92,0.98)	<0.001
p for trend		<0.001		0.02		0.03
	Cardiavascular disease mortality					
	Crude model		Model 1		Model 2	
ALI	95%CI	P	95%CI	P	95%CI	P
Q1	ref		ref		ref	
Q2	0.21(0.12,0.37)	<0.001	0.26(0.15,0.44)	<0.001	0.25(0.14,0.43)	<0.001
Q3	0.24(0.14,0.43)	<0.001	0.31(0.16,0.60)	<0.001	0.35(0.17,0.68)	0.002
Q4	0.35(0.15,0.83)	0.02	0.52(0.19,0.73)	0.01	0.56(0.18,0.71)	0.01
Per 10U increment	0.78(0.66,0.91)	<0.001	0.88(0.76, 0.93)	<0.001	0.92(0.86, 0.96)	<0.001
p for trend		0.01		<0.001		<0.001
	Respiratory mortality					
	Crude model		Model 1		Model 2	
ALI	95%CI	P	95%CI	P	95%CI	P
Q1	ref		ref		ref	
Q2	0.29(0.13,0.63)	0.002	0.33(0.15,1.21)	0.11	0.29(0.12, 0.67)	0.004
Q3	0.41(0.19,0.90)	0.03	0.49(0.22,1.12)	0.09	0.52(0.23, 0.77)	0.11
Q4	0.22(0.08,0.62)	0.004	0.32(0.11,0.91)	0.03	0.31(0.10, 0.93)	0.04
Per 10U increment	0.96(0.94,0.98)	<0.001	0.84(0.73,0.95)	<0.001	0.94(0.91,0.97)	<0.001
p for trend		<0.001		<0.001		0.02

ALI, advanced lung cancer inflammation index; CIAD, chronic inflammatory airway diseases; Q1, Quartile 1; Q2, Quartile 2; Q3, Quartile 3; Q4, Quartile 4; ref, reference; HR, hazard ratios; CI, confidence interval.

Crude model was not adjusted for any covariates.

Model 1 was adjusted for gender, age, and ethnicity.

Model 2 was adjusted for Model 1 plus BMI, alcohol user, smoking status, DM, CVD and hypertension.

Model 3 was adjusted for Model 2 plus education level, family income-to-poverty ratio, smoking and drinking status, marital status, BMI, and medical histories of hypertension, cardiovascular disease and diabetes.

Monitoring the dynamic changes of ALI is essential for predicting outcomes in CIAD patients. A 10-unit increase in ALI was linked to a 5% decrease in all-cause mortality, an 8% decrease in cardiovascular disease mortality, and a 6% decrease in respiratory disease mortality, after adjusting for all variables. The detailed results were displayed in **Table 2**.

### Non-linear relationships

**Figure 3:**
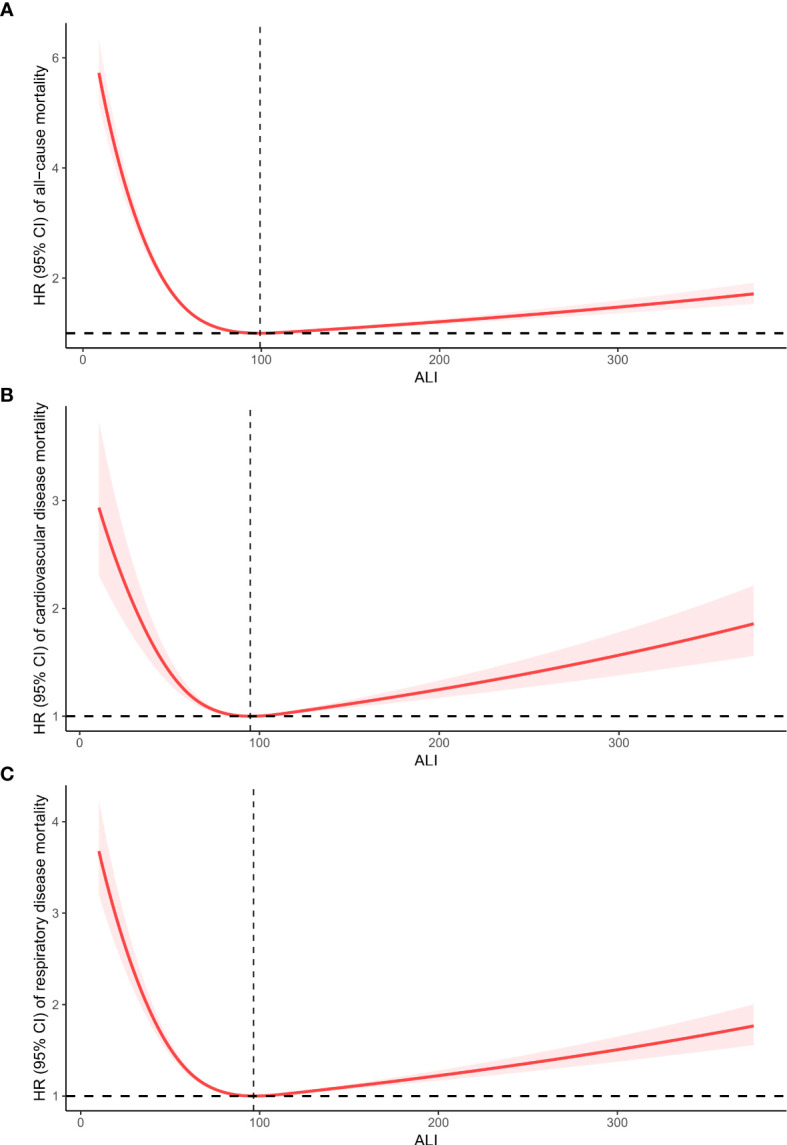
Restricted cubic spline analyses the relationship of advanced lung cancer inflammation index (ALI) levels and the risk of all-cause **(A)**, and cardiovascular diseases **(B)** and respiratory diseases mortality **(C)** in adults with chronic inflammatory airway diseases (CIAD). Adjusted for gender(female or male), age (<40, 40–60 or >60) and race (Mexican American, non-Hispanic black, non-Hispanic white or others); education level(completed high school, less than high school or more than high school), family income-to-poverty ratio(<1.3, 1.3-3.5 or >3.5), smoking status(never smoker, former smoker, or current smoker), drinking status (Current heavier drinker, Current light/moderate drinker, former drinker or never drinker), marital status(married/living with partner, never married or widowed/divorced), BMI(<25.0, 25.0-29.9 or >30.0 kg/m2), and histories of hypertension(yes or no), hyperlipidemia(yes or no), cardiovascular disease(yes or no) and diabetes(yes or no).

Upon accounting for all covariates (Model 2), the RCS analysis was utilized to display the connection between ALI and mortality from all causes and specific causes in CIAD adults. The overall pattern demonstrates a nonlinear correlation between ALI and deaths from all causes, with a inflection point at 99 (p for nonlinearity <0.0001) (**Figure 3A**). A similar J-shaped association was observed in mortality due to cardiovascular and respiratory diseases, with turning points at 94 (p for nonlinearity=0.0012) and 96 (p for nonlinearity <0.0001) (**Figures 3B, C**) respectively.

For ALI values below 99, a 10-unit rise in ALI was linked to a 14% reduction in the risk of all-cause mortality (HR: 0.86, 95% CI:0.81-0.92, Ptrend=0.01). Conversely, if ALI exceeded 99, a 10-unit increase in ALI resulted in a 3% rise in the risk of all-cause mortality (HR: 1.03, 95% CI:1.01-1.06, Ptrend=0.02). For ALI values below 94, a 10-unit increase in ALI was associated with a 10% decrease in the risk of cardiovascular diseases mortality (HR: 0.90, 95% CI:0.83-0.96, Ptrend=0.001). Nevertheless, if ALI surpassed 94, a 10-unit rise in ALI led to a 4% increase in the risk of cardiovascular diseases mortality (HR: 1.04, 95% CI:1.02-1.07, Ptrend=0.01). Similarly, for ALI values below 96, a 10-unit increase in ALI was linked to a 13% decrease in the risk of respiratory diseases mortality (HR: 0.87, 95% CI:0.82-0.97, Ptrend=0.03). On the other hand, if ALI exceeded 96, a 10-unit increase in ALI resulted in a 4% increase in the risk of respiratory diseases mortality (HR: 1.04, 95% CI:1.01-1.05, Ptrend=0.02). The summarized details are provided in **Table 3**.

**Table 3:** Threshold effect analysis of ALI on all-cause and cause-specific mortality in patients with CIAD from the NHANES 2013–2018 cohort.

	All-cause mortality	
ALI	Per 10U increment	P
<99	0.86(0.81- 0.92)	0.01
>99	1.03(1.01-1.06)	0.02
Cardiovascular disease mortality		
ALI	Per 10U increment	P
<94	0.90(0.83-0.96)	0.001
>94	1.04(1.02-1.07)	0.01
Respiratory disease mortality		
ALI	Per 10U increment	P
<96	0.87(0.82-0.97)	0.03
>96	1.04(1.01-1.05)	0.02

ALI, advanced lung cancer inflammation index; CIAD, chronic inflammatory airway diseases

### Subgroup and sensitivity analyses

The results displayed in **Table 4** demonstrated a significant connection between the ALI and overall mortality across different subgroups. Specifically, this relationship was notably apparent in individuals aged 60 years and above, females or males, non-Hispanic black or Mexican American, those with married/living with partner, individuals with more than high school, those with current or former smoking, former or never alcohol use, and those without hypertension and CVD or with a history of diabetes, with a statistically significant trend (p < 0.05). No significant interactions were observed between ALI and various subgroup variables in relation to all-cause mortality in patients with CIAD (all p for interaction > 0.05) (**Table 4**). Further subgroup analyses on cause-specific mortality can be found in **Supplementary Tables S1A, B**, with no significant interactions revealed in either respiratory or cardiovascular diseases mortality.

**Table 4:** Subgroup analysis of the association between quartiles of ALI and all cause mortality in patients with CIAD from the NHANES 2013–2018 cohort.

	Q1	Q2	Q3	Q4	p for trend	p for interaction
**Age**						0.39
20–40	ref	0.54(0.40,0.70)	0.79(0.59,1.20)	0.82(0.62,1.06)	0.55	
40–60	ref	0.53(0.45,0.61)	0.54(0.44,1.12)	0.86(0.67,1.17)	0.31	
>60	ref	0.45(0.37,0.75)	0.68(0.54,0.85)	0.64(0.51,0.79)	<0.001	
**Sex**						0.94
Female	ref	0.83(0.64,1.06)	0.76(0.60,0.96)	0.71(0.59,0.79)	0.01	
Male	ref	0.79(0.62,1.01)	0.65(0.53,0.80)	0.62(0.52,0.83)	<0.001	
**Family PIR**						0.16
<1.3	ref	0.72(0.57,0.91)	0.73(0.54,0.98)	0.77(0.60,1.19)	0.23	
1.3-3.5	ref	0.71(0.65,0.87)	0.61(0.76,0.81)	0.69(0.48,1.14)	0.10	
>3.5	ref	0.78(0.59,1.03)	0.73(0.64,1.12)	0.75(0.66,1.33)	0.08	
**Race**						0.64
non-Hispanic white	ref	0.62(0.35,1.09)	0.47(0.37,0.75)	0.56(0.35,1.09)	0.09	
non-Hispanic black	ref	0.94(0.72,1.23)	0.81(0.62,0.95)	0.79(0.56,0.92)	0.01	
Mexican American	ref	0.84(0.68,0.95)	0.72(0.58,0.90)	0.71(0.56,0.90)	0.01	
Others	ref	0.64(0.46,0.90)	0.65(0.48,0.87)	0.52(0.36,1.17)	0.59	
**Marital status**						0.51
Married/living with partner	ref	0.74(0.57,0.87)	0.73(0.58,0.93)	0.65(0.49,0.85)	0.01	
Widowed/divorced	ref	0.85(0.66,1.18)	0.90(0.68,1.28)	0.87(0.64,1.19)	0.37	
Never married	ref	1.30(0.71,1.59)	0.54(0.39,0.75)	0.75(0.53,1.17)	0.06	
**Education level**						0.21
Completed high school	ref	1.01(0.73,1.23)	0.93(0.77,1.35)	0.65(0.45,1.28)	0.49	
Less than high school	ref	0.81(0.66,1.22)	0.43(0.31,0.60)	0.66(0.54,1.13)	0.33	
More than high school	ref	0.75(0.61,0.86)	0.70(0.57,0.87)	0.75(0.59,0.84)	0.03	
**Smoking status**						0.28
Now	ref	0.95(0.68,1.32)	0.75(0.63,1.06)	0.61(0.43,0.84)	0.004	
Former	ref	0.76(0.57,1.01)	0.77(0.67,1.03)	0.53(0.30,0.63)	<0.001	
Never	ref	0.89(0.67,1.27)	0.82(0.65,1.12)	0.95(0.83,1.43)	0.58	
**Drinking status**						0.44
Current heavier drinker	ref	0.81(0.53,1.24)	0.78(0.63,0.88)	0.79(0.53,1.01)	0.14	
Current light/moderate drinker	ref	0.87(0.68,0.93)	0.80(0.71,0.89)	0.87(0.61,1.21)	0.30	
Former	ref	0.94(0.65,0.98)	0.81(0.50,0.92)	0.77(0.71,0.88)	0.001	
Never	ref	0.89(0.50,0.99)	0.72(0.45,0.84)	0.79(0.61,0.92)	0.01	
**DM**						0.17
No	ref	0.82(0.69,0.97)	0.70(0.58,0.83)	0.72(0.59,1.18)	0.76	
Yes	ref	0.83(0.60,1.15)	0.82(0.54,1.25)	0.60(0.40,0.89)	0.04	
**CVD**						0.58
No	ref	1.00(0.68,1.47)	0.89(0.56,1.42)	0.46(0.27,0.77)	0.02	
Yes	ref	0.83(0.71,0.97)	0.73(0.62,0.86)	0.77(0.64,1.23)	0.22	
**Hypertension**						0.29
No	ref	0.94(0.74,1.19)	0.80(0.61,1.05)	0.72(0.54,0.97)	0.03	
Yes	ref	0.73(0.57,0.93)	0.66(0.52,0.83)	0.69(0.55,1.27)	0.06	

ALI, advanced lung cancer inflammation index; CIAD, chronic inflammatory airway diseases; PIR, family poverty-to-income ratio; DM, diabetes mellitus; CVD, cardiovascular disease; Q1, Quartile 1; Q2, Quartile 2; Q3, Quartile 3; Q4, Quartile 4.

Moreover, we carried out sensitivity analyses by excluding individuals who died within 2 years of the follow-up period and re-conducted the Cox regression analysis. This reassessment validated the existence of the previously established correlation (see **Supplementary Tables S2**). Notably, after removing participants under 45 years old, our study’s conclusions remained unchanged (see **Supplementary Tables S3**). Furthermore, upon excluding individuals with a history of cancer at the beginning of the study, we also identified significant associations between higher ALI levels and decreased risks of overall mortality, as well as cardiovascular or respiratory diseases mortality among CIAD patients (see **Supplementary Tables S4**). These results suggest that the findings are reliable, consistent, and have broad clinical relevance.

## Discussion

This study was the first to investigate how ALI levels are connected to all-cause and cause-specific mortality in CIAD patients, using a comprehensive and publicly available representative cohort. The Cox model, adjusted for various factors, indicated that an increase in ALI was linked to a reduced risk of all-cause mortality, as well as mortality from cardiovascular and respiratory diseases among CIAD patients. Results from RCS analysis revealed that the lowest risks for all-cause mortality, cardiovascular diseases, and respiratory diseases mortality occurred at ALI levels of 99, 94, and 96, respectively. A non-linear association in the shape of an inverse J curve was observed between ALI and mortality from all causes, cardiovascular disease, and respiratory disease among CIAD participants. Using the inflection point as the boundary, ALI exhibits a shielding influence on the left-hand side. An increase of 10 units in ALI was associated with a 14% decrease in all-cause mortality risk, and a 10% and 13% decrease in cardiovascular diseases and respiratory diseases mortality risk, respectively. On the flip side, the risk of mortality increased by 3%, 4%, and 4% for all-cause, cardiovascular, and respiratory diseases mortality, respectively. The findings were consistent across sociodemographic and prior disease-related subgroups, and remained stable in sensitivity analyses. These results suggest that ALI, which combines information on inflammation and nutritional status, could be a useful tool for evaluating mortality risk in CIAD patients. Additionally, the inflection point in the J-shaped relationship represents the ALI value with the lowest risk of death, aiding in the accurate identification of mortality risk from all causes and cardiovascular or respiratory diseases in CIAD patients, and facilitating the development of personalized treatment plans in clinical settings.

Chronic inflammation has been linked to a range of long-term health conditions, including diabetes, various types of cancer, heart disease, and respiratory disorders (32). In the lungs, ongoing inflammation in the respiratory tract is a key factor in the development and progression of diseases such as chronic obstructive pulmonary disease (COPD) and asthma (33, 34). Prolonged inflammation is marked by the activation of inflammatory pathways and the breakdown of tissues. Asthma is characterized by environmental triggers that activate dendritic cells and airway epithelial cells, leading to the initiation of a Th2 immune response (35, 36). This cascade leads to the discharge of signaling molecules and cytokines from mast cells, basophils, and neutrophils, which eventually enhances the ability of eosinophil adhesion (37, 38). Several studies have shown that higher levels of inflammatory markers in the blood, including white blood cell count, eosinophil count, and lymphocyte count, are associated with more severe asthma symptoms (36, 39). COPD, a progressive inflammatory lung disease, is closely tied to smoking (40). Exposure to cigarette smoke leads to persistent and increased inflammation and dysregulation of the immune system in the lungs, which is a key factor in the occurrence and progression of COPD (41). In the context of COPD, alveolar macrophages are central players in the perpetuation of inflammation in COPD, as they can secrete chemokines that attract neutrophils and lymphocytes, ultimately leading to the accumulation of these cells in the lining of the airways (42).

Chronic inflammation can speed up the deterioration of proteins, resulting in lower levels of albumin. Furthermore, inflammation can trigger insulin resistance, reduce appetite, and interfere with nutrient absorption, ultimately causing weight loss (43, 44). Previous studies have indicated a strong connection existed between the nutritional status of patients with CIAD and their prognosis (17, 18). The ALI is a measure that combines serum albumin, body mass index, and the inflammatory marker NLR and evaluates both the inflammatory response and nutritional status of the individuals. What sets ALI apart from other indices is its inclusion of not only NLR and albumin but also BMI to assess nutritional well-being. A low body mass index is a recognized indicator of malnutrition (45), and preoperative low BMI is an independent predictor of prognosis as a nutritional parameter in patients with gastric cancer (46). Furthermore, preoperative albumin levels have also been identified as a prognostic indicator in gastric cancer patients, with low albumin being the most accurate predictor of short-term prognosis (47). By evaluating inflammation and nutritional status, ALI can accurately predict disease outcomes. The prognostic predictive ability of ALI has been validated in various diseases, both oncologic and non-oncologic, on a large scale. ALI has shown promise in predicting outcomes across various conditions such as lung cancer (20, 21), gastric cancer (22), diabetes (23), heart failure (24), and B-cell lymphoma (25). However, the relationship between ALI and mortality risk in CIAD patients has not yet been established. Our research has revealed a significant correlation between elevated ALI and reduced all-cause mortality, as well as mortality from cardiovascular and respiratory diseases, in CIAD patients. These results were consistent across multiple subgroup analyses and sensitivity analyses, highlighting ALI as a reliable prognostic indicator for CIAD patients with high robustness.

A non-linear, inverse J-shaped relationship was initially observed between ALI and all-cause and cause-specific mortality among patients with CIAD. This relationship can be explained from three key perspectives. Firstly, the NLR serves as a proxy for the body’s immune-inflammatory response, with high neutrophil counts indicating non-specific inflammation and low lymphocyte counts suggesting a relative deficiency of immune regulation (48). Therefore, elevated NLR levels could reflect the immune system’s functional state during chronic inflammation (49). Ke and colleagues found a strong link between inflammatory markers (NLR) obtained from complete blood cell counts and an increased risk of death from all causes and respiratory diseases in adults with asthma (50). In a prospective study, Lee and team discovered that the NLR was a dependable indicator for predicting COPD exacerbations and subsequent hospitalizations due to respiratory issues in individuals with COPD (51). Paliogiannis and others examined previous studies and highlighted the usefulness of the NLR as a direct and valuable predictor for forecasting acute exacerbations of COPD and mortality (52). The average NLR in the general population ranges from 1.65 to 2.11 (53, 54), whereas in this study, CIAD patients had an average NLR of 2.53. Leukocytes that have been activated release reactive oxygen species through neutrophils and cytokines, contributing to systemic inflammation (55). All the above evidence indicated that individuals with higher NLR have a worse prognosis in cases of CIAD. Our research shows that as the groups progressed from the first quartile (Q1) to the fourth quartile (Q4), NLR levels consistently decreased, resulting in a decrease in both all-cause and cause-specific mortality rates. Therefore, in the composite index ALI, we hypothesized that a decreased NLR was primarily responsible for the sustained reduction in both all-cause and cause-specific mortality rates among patients with CIAD.

Secondly, albumin was commonly utilized as a marker for assessing nutritional status. Previous studies have demonstrated a link between lower albumin levels and increased occurrences of complications in CIAD patients (17, 18). It is important to note that albumin has anti-inflammatory properties. Individuals with high albumin showed significant reduction in pro-inflammatory cytokines like TNF and CRP compared to the individuals with low albumin level (56). In essence, higher albumin level not only decreased the likelihood of CIAD complications, thereby improving the prognosis, but also contributed to a favorable prognosis due to its anti-inflammatory properties. The study observed a gradual increase in albumin levels from group Q1 to Q4, accompanied by a progressive decrease in mortality from all causes, as well as cardiovascular and respiratory diseases. As a result, we hypothesized that in the composite index ALI, higher albumin levels primarily served to a consistent reduction in the risk of all-cause and cause-specific mortality among patients with CIAD.

Furthermore, BMI was another commonly employed measure for evaluating nutritional status. A BMI of 30 or higher was used to classify individuals as obese. Researches have consistently shown that obesity is an independent risk factor for chronic respiratory conditions, including asthma and chronic obstructive pulmonary disease (57, 58). However, the relationship between BMI and outcomes for patients with CIAD is complex and has been the subject of debate. Some studies have found that obesity may actually be protective against mortality in COPD patients, a phenomenon known as the “obesity paradox” (59, 60). Nevertheless, the links between obesity and mortality in individuals with COPD and asthma have also yielded mixed and conflicting results (61). Several theories exist to explain the obesity paradox, yet a consensus is lacking. One of the most accepted explanations is that current obesity indicators has limitation in distinguishing between different types of body composition, including fat mass and muscle mass (60, 62). Another idea points to the impact of confounding factors like smoking status, with studies indicating that the obesity paradox is absent in non-smokers (63). Finally, many studies ignore potential biases related to obesity levels. Several researches indicated that the connection between BMI and CIAD prognosis was more complex than a straightforward linear relationship. A meta-analysis of various studies showed that there was a non-linear dose-response correlation between BMI and mortality among patients with COPD (64). At a BMI of 30 kg/m2, the mortality rate was at its lowest, while a BMI exceeding 32 kg/m2 showed no correlation with mortality rates. Similarly, another study found a non-linear relationship between BMI and all-cause mortality in CIAD patients, with an inflection value of BMI=32.4 kg/m2 (65). The lowest mortality rate for CIAD patients was observed at a BMI of 32.4 kg/m2. For BMI ≤ 32.4 kg/m2, all-cause mortality decreased as BMI increased. However, for BMI > 32.4 kg/m2, there was no observed link between BMI and all-cause mortality (65). Moreover, individuals with obesity often experience an elevated count of white blood cells in their peripheral blood, which can trigger heightened inflammatory responses and release more pro-inflammatory mediators (66). The inflammatory pathways shared by both diseases may serve as a key connecting links between them. Furthermore, obesity influences the ventilatory mechanics of individuals, which could be a crucial factor in the relationship between CIAD and obesity (67). Given these findings, we contended that BMI played a significant role in the inverse J-shaped relationship between ALI and all-cause and cause-specific mortality among CIAD patients.

The current study has the following key strengths. Firstly, the data utilized in this study sourced from a large public database that was nationally representative, guaranteeing its credibility, reliability, and representativeness. Secondly, we employed a range of statistical analysis methods to minimize interference from potential confounding variables on our results. These methods included multivariable adjusted Cox analysis, stratified analysis, interaction analysis and sensitivity analyses. Finally, ALI served as a comprehensive index that was both easy to derive and compute, making it highly suitable for clinical applications.

The current study has limitations that should be noted. Firstly, reliance on self-reported data to diagnose CIAD and a lack of information on treatment details may have influenced the results. Additionally, despite our efforts to account for multiple factors that could introduce bias in this study, there might still be unknown confounding variables affecting the outcomes. Secondly, due to the observational nature of the research, establishing a definitive causal relationship between ALI and mortality in CIAD patients was not feasible. Thirdly, the ALI indicator utilized in the study was derived from one-time complete blood count (CBC) parameters and serum albumin levels, potentially not reflecting the overall status of participants and introducing bias. Lastly, it is essential to acknowledge that the study was conducted among CIAD patients in the US, with a diverse sample of participants including 274 Mexican Americans, 637 Non-Hispanic Black individuals, 1,334 Non-Hispanic White individuals, and 639 individuals of other races. However, generalizing the findings to other populations may be limited. Future research should consider larger sample sizes, a prospective study design, and comprehensive adjustments for factors to better understand the causal mechanisms and confirm the impact of ALI on CIAD.

## Conclusions

Briefly, our study revealed a novel association between elevated ALI level and reduced all-cause and cause-specific mortality risk in patients with CIAD. Notably, the relationship between ALI and mortality rates from all cause, as well as cardiovascular and respiratory diseases, exhibited an inverse J-shaped curve, with optimal ALI levels corresponding to the lowest mortality risk at 99, 94, and 96, respectively. These findings underscore the importance of maintaining optimal ALI levels within a specific range to promote long-term survival in CIAD patients, which can be achieved through measures such as weight management, normalizing albumin levels, and anti-inflammatory therapies. Furthermore, the dynamic changes in ALI over time also can help clinicians establish personalized ALI standards to maximize the long-term outcomes for CIAD patients.

## Data Availability

The original contributions presented in the study are included in the article/**Supplementary Material**. Further inquiries can be directed to the corresponding author.
