# Supplementary material for: Relationship between advanced lung cancer inflammation index and all-cause and cause-specific mortality among chronic inflammatory airway diseases patients: a population-based study
Source: Front Immunol. 2025 May 15;16:1585927. doi: 10.3389/fimmu.2025.1585927 (PMC12119279; doi:10.3389/fimmu.2025.1585927)
Supplement: Supplementary file 1 [file Table1.docx]

**Relationship between Advanced lung cancer inflammation index and all-cause and cause-specific mortality among Chronic Inflammatory Airway Diseases patients: a population-based study**

**[Supplementary Tables S1A.](#_Toc88427342)** [Subgroup analysis of the association between quartiles of ALI and cardiovascular disease mortality in patients with CIAD from the NHANES 2013–2018 cohort.](#_Toc88427342).

**[Supplementary Tables S1B.](#_Toc88427343)** Subgroup analysis of the association between quartiles of ALI and respiratory disease mortality in patients with CIAD from the NHANES 2013–2018 cohort.

**[Supplementary Tables S2.](#_Toc88427343)** Relationships of ALI with all-cause and cause-specific mortality in patients with CIAD from the NHANES 2013–2018 cohort (Excluding Participants who Died within Two Years)

**[Supplementary Tables S3.](#_Toc88427343)** Relationships of ALI with all-cause and cause-specific mortality in patients with CIAD from the NHANES 2013–2018 cohort (Excluding Participants under the age of 45)

**[Supplementary Tables S4.](#_Toc88427343)** Relationships of ALI with all-cause and cause-specific mortality in patients with CIAD from the NHANES 2013–2018 cohort (Excluding Participants with a history of cancer at baseline)
